# Nonlocal Free Vibration of Embedded Short-Fiber-Reinforced Nano-/Micro-Rods with Deformable Boundary Conditions

**DOI:** 10.3390/ma15196803

**Published:** 2022-09-30

**Authors:** Ömer Civalek, Büşra Uzun, Mustafa Özgür Yaylı

**Affiliations:** 1 Civil Engineering Department, Akdeniz University, 07058 Antalya, Turkey; 2 Research Center for Interneural Computing, China Medical University, Taichung 40447, Taiwan; 3 Division of Mechanics, Civil Engineering Department, Bursa Uludag University, 16059 Bursa, Turkey

**Keywords:** axial vibration, short-fiber-reinforced, Fourier series, nonlocal elasticity

## Abstract

An efficient eigenvalue algorithm is developed for the axial vibration analysis of embedded short-fiber-reinforced micro-/nano-composite rods under arbitrary boundary conditions. In the formulation, nonlocal elasticity theory is used to capture the size effect, and the deformable boundary conditions at the ends are simulated using two elastic springs in the axial direction. In addition, to determine the reinforcing effect of restrained nano-/micro-rods, a new system of linear equations with the concept of the infinite power series is presented. After performing the mathematical processes known as Fourier sine series, Stokes’ transformation and successive integration, we finally obtain a coefficient matrix in terms of infinite series for various rigid or deformable boundary conditions. Some accurate eigenvalue solutions of the free axial vibration frequencies of the short-fiber-reinforced micro-/nano-composite rods with and without being restrained by the means of elastic springs are given to show the performance of the present method. The presence of the elastic spring boundary conditions changes the axial vibration frequencies and corresponding mode shapes.

## 1. Introduction

Due to their unique qualities, composite materials have garnered increasing attention over the past few decades. Composites are materials formed from at least two different components with different chemical and physical properties. These special materials have a more advanced structure than their constituent components thanks to the combination of different properties.

There are various types of composite materials used in different applications in the field of engineering. They can be constituted in different forms in accordance with the intended use. One of these is short-fiber-reinforced composite materials. These materials are formed by placing fibers of short length into a matrix in various arrangements.

Recently, studies involving the analysis of reinforced structures have gained momentum. Fiber-reinforced [1,2], carbon nanotube-reinforced [3,4,5,6,7,8,9,10] and graphene platelet-reinforced [11,12,13,14,15] structures can be found in the recent literature. However, other composite structures like functionally graded [16,17,18,19,20], sandwich [21,22] and porous functionally graded structures [23,24,25,26,27,28] have also attracted considerable interest. As can be seen, composite structures have attracted attention not only at a macro scale but also at nano and micro scales. While analyzing structures at nano and micro scales, we see that many of them are presented with theories based on the size effect. When we look at the governing equations represented by these theories, it is understood that they contain one or more parameters in addition to the classical constants. These parameters, generally called small-scale parameters, make it possible to investigate the size effect. Researchers working on the analysis of various very small-scale nano- and micro-structures have used these size effect theories, such as doublet mechanics theory [29,30,31,32,33], modified couple stress theory [34,35,36,37,38,39], nonlocal elasticity theory [1,40,41,42,43,44,45], nonlocal strain gradient theory [46,47,48,49] and strain gradient theory [50,51,52,53]. Researchers use a variety of techniques to analyze an element or structure, such as a rod, beam, plate, frame, etc., whether at the macro, nano, or micro level. Some of these techniques are: the finite element method [1,33,41,42,54,55], artificial neural networks [56], the Laplace transform [57], Stokes’ transformation [18,28,43,45], the perturbation technique [5] and the Chebyshev–Ritz method [19]. This is the first work to investigate the longitudinal vibration behavior of short-fiber-reinforced micro-/nano-rods embedded in an elastic medium via Fourier sine series with Stokes’ transformation.

This paper presents the free axial vibrational response of a restrained and size-dependent micro- and nano-scale rod embedded in an elastic medium based on Eringen’s nonlocal elasticity theory [58]. The size dependency of the material characteristics are modeled according to the short-fiber-reinforced micro- and nano-composite rods. In addition, displacements at the ends are defined based on classical rod theory. This paper presents for the first time a solution based on the Stokes’ transformation and Fourier sine series for the axial vibration of short-fiber-reinforced nano-/micro-rods with arbitrary boundary conditions in the presence of an elastic medium. The contribution of this work is that it provides an approach to study the effect of both an elastic medium and arbitrary boundary conditions on the axial vibration of short-fiber-reinforced micro-/nano-rods. Fourier sine series are also utilized to define the axial deflection function. Nonlocal force boundary conditions are utilized to derive the systems of linear equations for specifying the elastic foundation, nonlocal and short fiber parameters. The linear system of equations obtained is discretized with the help of Stokes’ transformation. A coefficient matrix and the corresponding eigenvalue problem is constructed for longitudinal dynamic analysis of the short-fiber-reinforced micro- and nano-composite rods under rigid or restrained boundary conditions. This coefficient matrix includes nonlocal parameter boundary conditions, a short fiber constant and an elastic foundation coefficient.

## 2. Nonlocal Elasticity

Nonlocal elasticity theory is the most preferred continuum mechanics approach for nano-sized structures. This continuum mechanics theory was introduced by Eringen [58]. In accordance with nonlocal elasticity theory, the stresses and strains of one location inside a structure are related to the stresses and strains of other locations that are close to the reference point.

For the axial vibration behavior of a composite micro-/nano-sized rod, the constitutive equation based on the nonlocal elasticity theory is written as follows [1]:

(1) $$\left[1-\mu \frac{\partial^{2}}{\partial x^{2}}\right]\tau_{xx}=E_{c}\varepsilon$$

where $\mu =e_{0}a^{2}$, $\tau_{xx}$ denotes the nonlocal stress, $E_{c}$ specifies the Young’s modulus of the composite, and ε represents the axial strain. In addition, $e_{0}a$ is called the nonlocal parameter. This nonlocal parameter allows us to investigate the size effect on the composite micro-/nano-rod. Furthermore, a defines the internal characteristic length and *e*_0_ specifies a material constant. The equation of motion for the longitudinal vibration of the composite micro- and nano-rods can be described by [44]:

(2) $$\frac{\partial N^{l}}{\partial x}+f=\rho_{c}A\frac{\partial^{2}u\left(x,t\right)}{\partial t^{2}}$$

in which $N^{l}$ denotes the axial force of local (classical) elasticity, f specifies the distributed axial force acting on the composite rod, $\rho_{c}$ represents the density of the composite, A is the cross-sectional area, $u\left(x,t\right)$ is the axial displacement and t is the time. In addition, the axial force of local elasticity $N^{l}$ can be defined as:

(3) $$N^{l}=\overset{}{\underset{A}{\int }}\sigma_{xx}dA$$

In Equation (3), $\sigma_{xx}$ is the component of local stress. By integrating Equation (1) with respect to the cross-sectional area of the composite, the following relation is obtained:

(4) $$N-\mu \frac{\partial^{2}N}{\partial t^{2}}=N^{l}$$

Here, N represents the axial force of nonlocal elasticity theory and is defined as:

(5) $$N=\overset{}{\underset{A}{\int }}\tau_{xx}dA$$

One can derive the equation of motion for longitudinal vibration of the composite micro- and nano-rods via Equations (2)–(5):

(6) $$E_{c}A\frac{\partial^{2}u\left(x,t\right)}{\partial x^{2}}+f-\mu \frac{\partial^{2}f}{\partial x^{2}}=\rho_{c}A\frac{\partial^{2}u\left(x,t\right)}{\partial t^{2}}-\mu \rho_{c}A\frac{\partial^{4}u\left(x,t\right)}{\partial x^{2}\partial t^{2}}$$

In this paper, the influence of an elastic medium on the longitudinal vibration frequencies of the short-fiber-reinforced composite micro- and nano-rods is investigated for the first time. For this purpose, the force based on the elastic medium is considered in the following form [44]:

(7) $$f=-ku\left(x,t\right)$$

In this study, the composite micro-/nano-rod is considered to be surrounded by an elastic medium. As expected, this elastic medium has a stiffness. In Equation (7), k denotes the stiffness of the elastic medium. One can derive the equation of motion for an embedded composite micro-/nano-rod by inserting Equation (7) into Equation (6) as follows:

(8) $$E_{c}A\frac{\partial^{2}u\left(x,t\right)}{\partial x^{2}}-ku\left(x,t\right)+\mu k\frac{\partial^{2}u\left(x,t\right)}{\partial x^{2}}-\rho_{c}A\frac{\partial^{2}u\left(x,t\right)}{\partial t^{2}}+\mu \rho_{c}A\frac{\partial^{4}u\left(x,t\right)}{\partial x^{2}\partial t^{2}}=0$$

It should be highlighted here that if the parameter μ is set to zero, the equation is simplified to the equation of the classical embedded composite rod as follows:

(9) $$E_{c}A\frac{\partial^{2}u\left(x,t\right)}{\partial x^{2}}-ku\left(x,t\right)-\rho_{c}A\frac{\partial^{2}u\left(x,t\right)}{\partial t^{2}}=0$$

The other point that should be highlighted here is that if the elastic medium stiffness k is set to zero, the equation is reduced to the equation of the un-embedded composite micro-/nano-rod as follows [1]:

(10) $$E_{c}A\frac{\partial^{2}u\left(x,t\right)}{\partial x^{2}}-\rho_{c}A\frac{\partial^{2}u\left(x,t\right)}{\partial t^{2}}+\mu \rho_{c}A\frac{\partial^{4}u\left(x,t\right)}{\partial x^{2}\partial t^{2}}=0$$

## 3. Material Properties of Short-Fiber-Reinforced Composite

**Figure 1:**
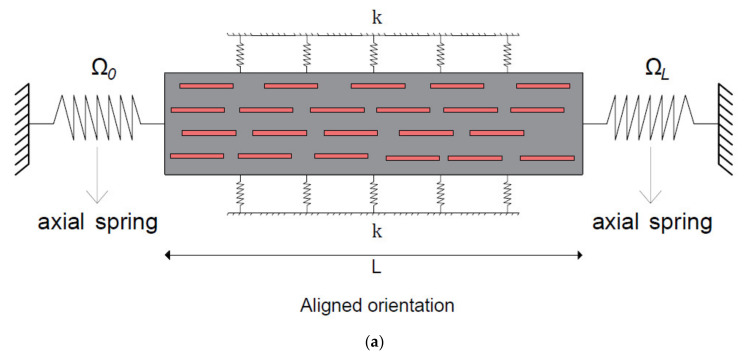

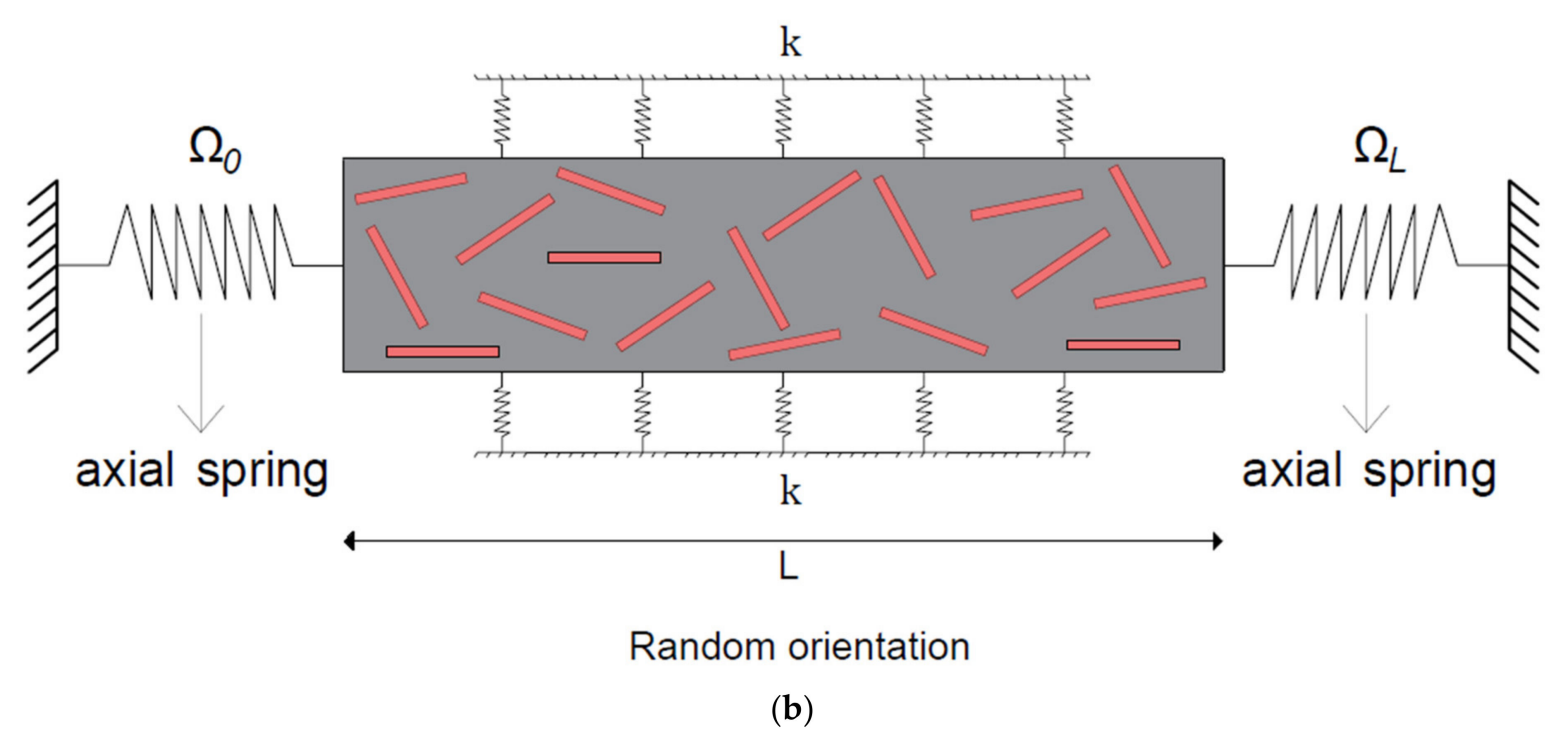
Illustrations of composite micro-/nano-rods with elastic springs: (**a**) aligned composite micro-/nano-rod; (**b**) randomly oriented composite micro-/nano-rod.

**Figure 2:**
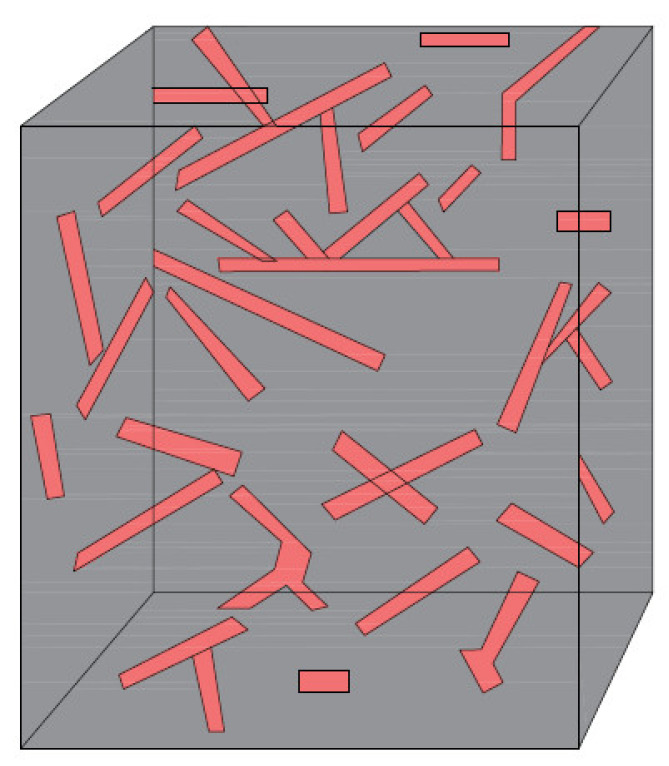
Illustration of a randomly oriented composite material.

**Figure 3:**
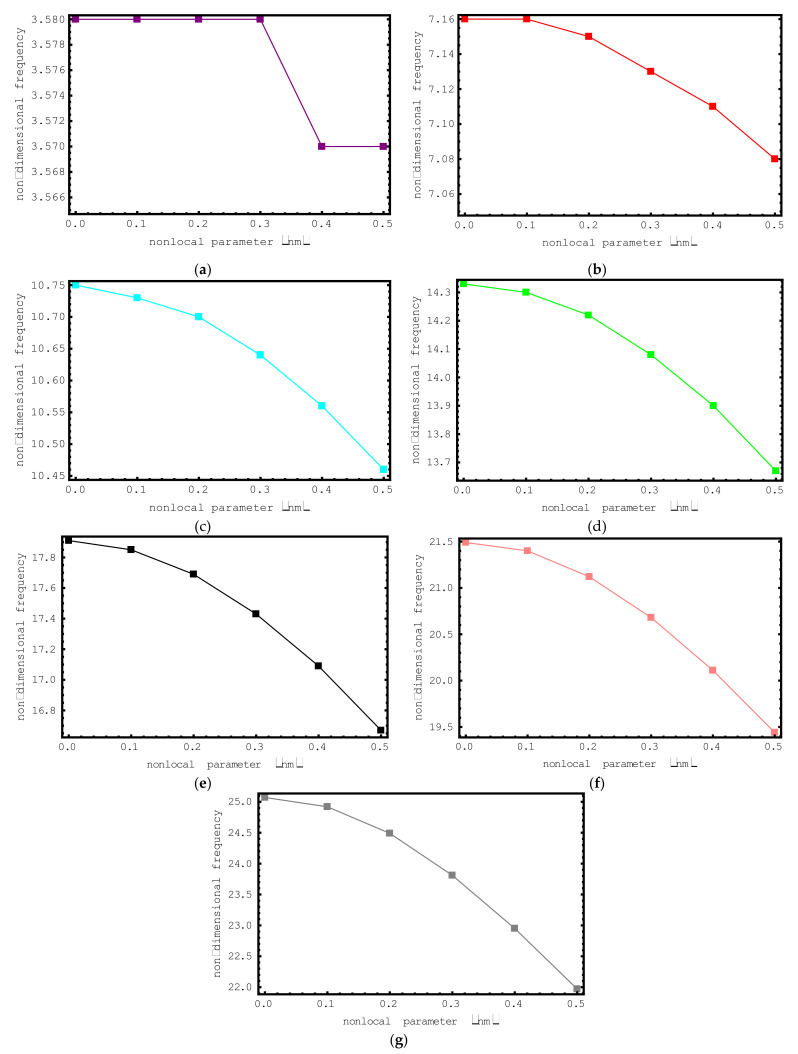
The variations of non-dimensional frequencies of short-fiber-reinforced composite nano-rods versus nonlocal parameter: (**a**) 1st mode (**b**) 2nd mode (**c**) 3rd mode (**d**) 4th mode (**e**) 5th mode (**f**) 6th mode (**g**) 7th mode.

In this study, the free longitudinal vibration behavior of the embedded short-fiber-reinforced composite micro-/nano-rod with deformable boundary conditions is investigated via Eringen’s nonlocal elasticity theory [58]. Furthermore, an aligned composite micro-/nano-rod and a randomly oriented composite micro-/nano-rod with elastic springs are shown in Figure 1 and Figure 2, respectively. In addition, an illustration of a randomly oriented composite material may be seen in Figure 3. As can be seen in the governing equation of embedded nano-rods given in Equation (10), Young’s modulus ($E_{c}$) and density ($\rho_{c}$) are properties of short-fiber-reinforced micro-/nano-rods that should be defined. In this section, these properties are described. This study adopts the Halpin–Tsai equations [59]. These simple and easy-to-use equations are quite reasonable as they give accurate predictions, as long as the fiber volume fraction does not approach one [60]. Via Halpin–Tsai equations, the longitudinal and transverse Young’s moduli of aligned short-fiber-reinforced composite materials are written as follows [60]:

(11) $$E_{L}=E_{m}\left(\frac{1+\left(\frac{2l}{d}\right)\eta_{L}V_{f}}{1-\eta_{L}V_{f}}\right)$$

(12) $$E_{T}=E_{m}\left(\frac{1+2\eta_{T}V_{f}}{1-\eta_{T}V_{f}}\right)$$

By arranging short fibers in a matrix in different ways, short-fiber-reinforced composite materials are formed. In the above equations, $E_{m}$ represents the Young’s modulus of the matrix, d is the diameter of the fiber, l denotes the length of the fiber and $V_{f}$ is the volume fraction of the fiber. In addition, $\eta_{L}$ and $\eta_{T}$ seen in Equations (11) and (12) are described as:

(13) $$\eta_{L}=\left(\frac{\frac{E_{f}}{E_{m}}-1}{\frac{E_{f}}{E_{m}}+2\left(\frac{l}{d}\right)}\right)$$

(14) $$\eta_{T}=\left(\frac{\frac{E_{f}}{E_{m}}-1}{\frac{E_{f}}{E_{m}}+2}\right)$$

in which $E_{f}$ specifies the Young’s modulus of the fiber of the composite. Moreover, the Young’s modulus of a randomly oriented short-fiber-reinforced composite is described as follows [60]:

(15) $$E_{c}=E_{random}=\frac{3}{8}E_{L}+\frac{5}{8}E_{T}$$

Lastly, the density of the short-fiber-reinforced composite should be defined. The density of the short-fiber-reinforced composite is given as follows [1]:

(16) $$\rho_{c}=\rho_{m}\left(1-V_{f}\right)+\rho_{f}V_{f}$$

In the above equation, $\rho_{c}$, $\rho_{m}$ and $\rho_{f}$ are the density of the short-fiber-reinforced composite, matrix and fiber, respectively. Short-fiber-reinforced composites include two different components, the matrix and the fibers, with different material properties. Depending on these components’ properties, the composite’s material properties are calculated with the equations given above.

## 4. Fourier Infinite Series with Stokes’ Transformation

In this section of the study, the adopted solution procedure is applied to the longitudinal vibration of embedded short-fiber-reinforced composite micro-/nano-rods restrained with axial elastic springs at both ends. Assuming harmonic vibrations, *u*(*x*, *t*) may be represented by:

(17) $$u\left(x,t\right)=\Psi \left(x\right)\cos\left(\omega t\right)$$

One can get the following expression by substituting Equation (17) into Equation (8):

(18) $$-E_{c}A\frac{d^{2}\Psi \left(x\right)}{dx^{2}}-k\Psi \left(x\right)-\mu k\frac{d^{2}\Psi \left(x\right)}{dx^{2}}-\rho_{c}A\omega^{2}\Psi \left(x\right)+\mu \rho_{c}A\omega^{2}\frac{d^{2}\Psi \left(x\right)}{dx^{2}}=0$$

in which *ω* specifies the natural frequency of the composite micro-/nano-rod in terms of rad/s and Ψ(*x*) defines the modal displacement function, and Ψ(*x*) can be written in three separate regions as below [43,45]:

(19) $$\Psi \left(x\right)=\Psi_{0}\;x=0,$$

(20) $$\Psi \left(x\right)=\Psi_{L}\;x=L,\;$$

(21) $$\Psi \left(x\right)=\sum_{j=1}^{\infty }H_{j}\sin\left(\frac{j\pi x}{L}\right)\;0<x<L$$

$H_{j}$ in Equation (21) is defined as:

(22) $$H_{j}=\frac{2}{L}\int_{0}^{L}\Psi \left(x\right)\sin\left(\frac{j\pi x}{L}\right)dx$$

The first derivative of Equation (21) leads to:

(23) $$\Psi^{\prime }\left(x\right)=\sum_{j=1}^{\infty }\frac{j\pi }{L}H_{j}\cos\left(\frac{j\pi x}{L}\right)$$

Furthermore, we can write Equation (23) as a Fourier cosine infinite series as follows:

(24) $$\Psi^{\prime }\left(x\right)=\frac{h_{0}}{L}+\sum_{j=1}^{\infty }h_{j}\cos\left(\frac{j\pi x}{L}\right)$$

The coefficients $h_{0}$ and $h_{j}$ can be expressed as:

(25) $$h_{0}=\frac{2}{L}\int_{0}^{L}\Psi^{\prime }\left(x\right)dx=\frac{2}{L}\left[\Psi \left(L\right)-\Psi \left(0\right)\right]$$

(26) $$h_{j}=\frac{2}{L}\int_{0}^{L}\Psi^{\prime }\left(x\right)\cos\left(\frac{j\pi x}{L}\right)\mathrm{dx}\;j=1,\;2,\;\ldots$$

If we apply integration by parts, we obtain the following expressions:

(27) $$h_{j}=\frac{2}{L}{\left[\Psi \left(x\right)\;\cos\left(\frac{j\pi x}{L}\right)\right]}_{0}^{L}+\frac{2}{L}\left[\frac{j\pi }{L}\int_{0}^{L}\Psi \left(x\right)\sin\left(\frac{j\pi x}{L}\right)dx\right]$$

(28) $$h_{j}=\frac{2}{L}\left[{\left(-1\right)}^{j}\Psi \left(L\right)-\Psi \left(0\right)\right]+\frac{j\pi }{L}H_{j}$$

To continue the mathematical steps, we should find the first two derivatives of the displacement function $\Psi \left(x\right)$. The first two derivatives of $\Psi \left(x\right)$ are calculated as [43,45]:

(29) $$\;\frac{d\Psi \left(x\right)}{dx}=\frac{\Psi_{L\;}-\Psi_{o\;}\;}{L}\;\sum_{j=1}^{\infty }\cos\left(\xi_{j}x\right)\left(\frac{2\left({\left(-1\right)}^{j}\Psi_{L}-\Psi_{0}\right)}{L}+\xi_{j}H_{j}\;\right)$$

(30) $$\frac{d^{2}\Psi \left(x\right)}{dx^{2}}=\;\;\sum_{j=1}^{\infty }\xi_{j}\sin\left(\xi_{j}x\right)\left(\frac{2\left({\left(-1\right)}^{j}\Psi_{L}-\Psi_{0}\right)}{L}+\xi_{j}H_{j}\;\right)$$

in which $\xi_{j}$ is defined as:

(31) $$\xi_{j}=\frac{j\pi }{L}$$

In this step of the solution, we should find the Fourier coefficient $H_{j}$. To find $H_{j}$, we substitute Equations (21), (30) and (31) into Equation (18):

(32) $$H_{j}=\frac{2j\pi \left(-\gamma^{2}\lambda^{2}+\gamma^{2}K+1\right)\left(\Omega_{0}-{\left(-1\right)}^{j}\Omega_{L}\right)}{-\lambda^{2}+K^{2}+\pi^{2}j^{2}\gamma^{2}K-\pi^{2}j^{2}\gamma^{2}\lambda^{2}+\pi^{2}j^{2}}$$

Here,

(33) $$\gamma^{2}=\frac{\mu }{L^{2}}$$

(34) $$K=\frac{kL^{2}}{E_{m}A}$$

(35) $$\lambda^{2}=\frac{\rho_{c}A\omega^{2}L^{2}}{E_{c}A}$$

The axial displacement for the axial vibration of a composite micro-/nano-sized rod embedded in an elastic medium yields:

(36) $$u\left(x,t\right)=\sum_{j=1}^{\infty }\frac{2j\pi \left(-\gamma^{2}\lambda^{2}+\gamma^{2}K+1\right)\left(\Omega_{0}-{\left(-1\right)}^{j}\Omega_{L}\right)}{-\lambda^{2}+K^{2}+\pi^{2}j^{2}\gamma^{2}K-\pi^{2}j^{2}\gamma^{2}\lambda^{2}+\pi^{2}j^{2}}\sin\left(\frac{j\pi x}{L}\right)\cos\left(\omega t\right)$$

The above equation is the more general axial displacement equation, consisting of the elastic medium effect and small size effect for a composite micro-/nano-rod.

## 5. Frequency Determinants for the Short-Fiber-Reinforced Micro-/Nano-Rods

In this section of the study, a number of eigenvalue problems for the various degenerated cases of short-fiber-reinforced micro-/nano-rods based on nonlocal elasticity are set up. Via these eigenvalue problems, the axial vibration frequencies of the short-fiber-reinforced micro-/nano-rods are found.

### 5.1. General Case

To obtain the free axial frequencies of embedded short-fiber-reinforced micro-/nano-rods, size-dependent boundary conditions should be written in terms of elastic axial springs at both ends.

(37) $$E_{c}A\frac{\partial u}{\partial x}+\mu \rho_{c}A\frac{\partial^{3}u}{\partial x\partial t^{2}}=\;\Omega_{0}\Psi_{0},\;\;\;\;\;\;\;\;\;\;\;\;\;\;\;\;\;x=0$$

(38) $$E_{c}A\frac{\partial u}{\partial x}+\mu \rho_{c}A\frac{\partial^{3}u}{\partial x\partial t^{2}}=\;\Omega_{L}\Psi_{L},\;\;\;\;\;\;\;\;\;\;\;\;\;\;\;\;\;x=L$$

In the above equations, $\Omega_{0}$ and $\Omega_{L}$ define the axial spring stiffnesses of the short-fiber-reinforced nano-rod. By inserting Equations (29) and (36) into Equations (37) and (38), the following two homogeneous equations are found:

(39) $$\left(\gamma^{2}\lambda^{2}-\bar{\Omega_{0}}-1+\overset{\infty }{\underset{j=1}{\sum }}\frac{2\left(-\gamma^{2}\lambda^{4}+\lambda^{2}+\gamma^{2}\lambda^{2}K-K\right)}{-\lambda^{2}+\pi^{2}j^{2}\gamma^{2}K+K-\pi^{2}j^{2}\gamma^{2}\lambda^{2}+\pi^{2}j^{2}}\right)\Psi_{0}+\left(-\gamma^{2}\lambda^{2}+1-\overset{\infty }{\underset{j=1}{\sum }}\frac{2{\left(-1\right)}^{j}\left(-\gamma^{2}\lambda^{4}+\lambda^{2}+\gamma^{2}\lambda^{2}K-K\right)}{-\lambda^{2}+\pi^{2}j^{2}\gamma^{2}K+K-\pi^{2}j^{2}\gamma^{2}\lambda^{2}+\pi^{2}j^{2}}\right)\Psi_{L}=0$$

(40) $$\left(-\gamma^{2}\lambda^{2}+1-\overset{\infty }{\underset{j=1}{\sum }}\frac{2{\left(-1\right)}^{j}\left(-\gamma^{2}\lambda^{4}+\lambda^{2}+\gamma^{2}\lambda^{2}K-K\right)}{-\lambda^{2}+\pi^{2}j^{2}\gamma^{2}K+K-\pi^{2}j^{2}\gamma^{2}\lambda^{2}+\pi^{2}j^{2}}\right)\Psi_{0}+\left(\gamma^{2}\lambda^{2}-\bar{\Omega_{L}}-1+\overset{\infty }{\underset{j=1}{\sum }}\frac{2\left(-\gamma^{2}\lambda^{4}+\lambda^{2}+\gamma^{2}\lambda^{2}K-K\right)}{-\lambda^{2}+\pi^{2}j^{2}\gamma^{2}K+K-\pi^{2}j^{2}\gamma^{2}\lambda^{2}+\pi^{2}j^{2}}\right)\Psi_{L}=0$$

In the above equations, $\bar{\Omega_{0}}$ and $\bar{\Omega_{L}}$ are the non-dimensional forms of the stiffnesses of axial springs and they are defined by:

(41) $$\bar{\Omega_{0}}=\frac{\Omega_{0}L}{E_{m}A}$$

(42) $$\bar{\Omega_{L}}=\frac{\Omega_{L}L}{E_{m}A}$$

Via Equations (39) and (40), the following eigenvalue problem is constructed to be resolved for the constants $\Psi_{0}$ and $\Psi_{L}$:

(43) $$\left[\begin{array}{cc} \Gamma_{11} & \Gamma_{12}\\ \Gamma_{21} & \Gamma_{22} \end{array}\right]\left[\begin{array}{c} \Psi_{0}\\ \Psi_{L} \end{array}\right]=0$$

The elements of the coefficient matrix are given as:

(44) $$\Gamma_{11}=\gamma^{2}\lambda^{2}-\bar{\Omega_{0}}-1+\overset{\infty }{\underset{j=1}{\sum }}\frac{2\left(-\gamma^{2}\lambda^{4}+\lambda^{2}+\gamma^{2}\lambda^{2}K-K\right)}{-\lambda^{2}+\pi^{2}j^{2}\gamma^{2}K+K-\pi^{2}j^{2}\gamma^{2}\lambda^{2}+\pi^{2}j^{2}}$$

(45) $$\Gamma_{12}=-\gamma^{2}\lambda^{2}+1-\overset{\infty }{\underset{j=1}{\sum }}\frac{2{\left(-1\right)}^{j}\left(-\gamma^{2}\lambda^{4}+\lambda^{2}+\gamma^{2}\lambda^{2}K-K\right)}{-\lambda^{2}+\pi^{2}j^{2}\gamma^{2}K+K-\pi^{2}j^{2}\gamma^{2}\lambda^{2}+\pi^{2}j^{2}}$$

(46) $$\Gamma_{21}=-\gamma^{2}\lambda^{2}+1-\overset{\infty }{\underset{j=1}{\sum }}\frac{2{\left(-1\right)}^{j}\left(-\gamma^{2}\lambda^{4}+\lambda^{2}+\gamma^{2}\lambda^{2}K-K\right)}{-\lambda^{2}+\pi^{2}j^{2}\gamma^{2}K+K-\pi^{2}j^{2}\gamma^{2}\lambda^{2}+\pi^{2}j^{2}}$$

(47) $$\Gamma_{22}=\gamma^{2}\lambda^{2}-\bar{\Omega_{L}}-1+\overset{\infty }{\underset{j=1}{\sum }}\frac{2\left(-\gamma^{2}\lambda^{4}+\lambda^{2}+\gamma^{2}\lambda^{2}K-K\right)}{-\lambda^{2}+\pi^{2}j^{2}\gamma^{2}K+K-\pi^{2}j^{2}\gamma^{2}\lambda^{2}+\pi^{2}j^{2}}$$

Free vibration frequencies of embedded short-fiber reinforced nano-rods are found by the eigenvalues of the coefficient matrix in Equation (43).

(48) $$\left|\Gamma_{\varphi \tau }\right|=0\;\left(\varphi ,\tau =1,2\right)$$

The above solution covers the impacts of the elastic medium, the nonlocal parameter and axial spring parameters. Nano-/micro-rods are one of the most important elements in various engineering applications. When these elements are used as a component, they need to be fixed to a place or another element. In theoretical studies of nano-/micro-rods, the fixation patterns are often investigated with the same idealized combinations. The analysis considers these combinations as clamped–clamped or clamped–free for rod elements. In these combinations, the boundary conditions are considered to be and investigated as fully rigid. On the other hand, during the realization of the engineering applications mentioned, it may not be possible to give full rigidity to the boundaries of the rods. This leads to a situation where the boundary conditions allow deformation contrary to what is assumed. This paper presents an approach to investigate the deformation-permitting boundary conditions of short-fiber-reinforced micro-/nano-rods. In applications where short-fiber-reinforced micro-/nano-rods are used or likely to be used and subjected to vibration, the dynamic behavior of these elements is important. With the solution approach presented in this study, inferences regarding the vibration behavior of short-fiber-reinforced micro-/nano-rods can be found for any desired boundary condition. For this purpose, it is sufficient to input the desired stiffness values of the axial springs attached to the ends of the micro-/nano-rod.

### 5.2. Without Elastic Medium Effect

To compute the free axial frequencies of short-fiber-reinforced micro-/nano-rods without the elastic medium effect, the non-dimensional elastic medium parameter in Equations (39) and (40) is set to zero. If we adjust the non-dimensional elastic medium parameter *K* in Equations (39) and (40) to zero, we obtain the following equations:

(49) $$\left(\gamma^{2}\lambda^{2}-\bar{\Omega_{0}}-1+\overset{\infty }{\underset{j=1}{\sum }}\frac{2\left(-\gamma^{2}\lambda^{4}+\lambda^{2}\right)}{-\lambda^{2}-\pi^{2}j^{2}\gamma^{2}\lambda^{2}+\pi^{2}j^{2}}\right)\Psi_{0}+\left(-\gamma^{2}\lambda^{2}+1-\overset{\infty }{\underset{j=1}{\sum }}\frac{2{\left(-1\right)}^{j}\left(-\gamma^{2}\lambda^{4}+\lambda^{2}\right)}{-\lambda^{2}-\pi^{2}j^{2}\gamma^{2}\lambda^{2}+\pi^{2}j^{2}}\right)\Psi_{L}=0$$

(50) $$\left(-\gamma^{2}\lambda^{2}+1-\overset{\infty }{\underset{j=1}{\sum }}\frac{2{\left(-1\right)}^{j}\left(-\gamma^{2}\lambda^{4}+\lambda^{2}\right)}{-\lambda^{2}-\pi^{2}j^{2}\gamma^{2}\lambda^{2}+\pi^{2}j^{2}}\right)\Psi_{0}+\left(\gamma^{2}\lambda^{2}-\bar{\Omega_{L}}-1+\overset{\infty }{\underset{j=1}{\sum }}\frac{2\left(-\gamma^{2}\lambda^{4}+\lambda^{2}\right)}{-\lambda^{2}-\pi^{2}j^{2}\gamma^{2}\lambda^{2}+\pi^{2}j^{2}}\right)\Psi_{L}=0$$

Thus, the eigenvalue problem to be obtained from the above two equations will be as follows:

(51) $$\left[\begin{array}{cc} \chi_{11} & \chi_{12}\\ \chi_{21} & \chi_{22} \end{array}\right]\left[\begin{array}{c} \Psi_{0}\\ \Psi_{L} \end{array}\right]=0$$

The elements of the coefficient matrix given above are given as:

(52) $$\chi_{11}=\gamma^{2}\lambda^{2}-\bar{\Omega_{0}}-1+\sum_{j=1}^{\infty }\frac{2\left(-\gamma^{2}\lambda^{4}+\lambda^{2}\right)}{-\lambda^{2}-\pi^{2}j^{2}\gamma^{2}\lambda^{2}+\pi^{2}j^{2}}$$

(53) $$\chi_{12}=-\gamma^{2}\lambda^{2}+1-\sum_{j=1}^{\infty }\frac{2{\left(-1\right)}^{j}\left(-\gamma^{2}\lambda^{4}+\lambda^{2}\right)}{-\lambda^{2}-\pi^{2}j^{2}\gamma^{2}\lambda^{2}+\pi^{2}j^{2}}$$

(54) $$\chi_{21}=-\gamma^{2}\lambda^{2}+1-\sum_{j=1}^{\infty }\frac{2{\left(-1\right)}^{j}\left(-\gamma^{2}\lambda^{4}+\lambda^{2}\right)}{-\lambda^{2}-\pi^{2}j^{2}\gamma^{2}\lambda^{2}+\pi^{2}j^{2}}$$

(55) $$\chi_{22}=\gamma^{2}\lambda^{2}-\bar{\Omega_{L}}-1+\sum_{j=1}^{\infty }\frac{2\left(-\gamma^{2}\lambda^{4}+\lambda^{2}\right)}{-\lambda^{2}-\pi^{2}j^{2}\gamma^{2}\lambda^{2}+\pi^{2}j^{2}}$$

The free vibration frequencies of embedded short-fiber-reinforced nano-rods are found by the eigenvalues of the coefficient matrix in Equation (51).

(56) $$\left|\chi_{\varphi \tau }\right|=0\;\left(\varphi ,\tau =1,2\right)$$

The above solution covers the influence of the nonlocal parameter and axial spring parameters.

### 5.3. Without Nonlocal Effect

To compute the free axial frequencies of embedded short-fiber-reinforced micro-/nano-rods without a size effect, the non-dimensional nonlocal parameter in Equations (39) and (40) is set to zero. If we adjust the non-dimensional nonlocal parameter *β* in Equations (39) and (40) to zero, we obtain the following equations:

(57) $$\left(-\bar{\Omega_{0}}-1+\overset{\infty }{\underset{j=1}{\sum }}\frac{2\left(\lambda^{2}-K\right)}{-\lambda^{2}+K+\pi^{2}j^{2}}\right)\Psi_{0}+\left(1-\overset{\infty }{\underset{j=1}{\sum }}\frac{2{\left(-1\right)}^{j}\left(+\lambda^{2}-K\right)}{-\lambda^{2}+K+\pi^{2}j^{2}}\right)\Psi_{L}=0$$

(58) $$\left(1-\overset{\infty }{\underset{j=1}{\sum }}\frac{2{\left(-1\right)}^{j}\left(\lambda^{2}-K\right)}{-\lambda^{2}+K+\pi^{2}j^{2}}\right)\Psi_{0}+\left(-\bar{\Omega_{L}}-1+\overset{\infty }{\underset{j=1}{\sum }}\frac{2\left(\lambda^{2}-K\right)}{-\lambda^{2}+K+\pi^{2}j^{2}}\right)\Psi_{L}=0$$

Thus, the eigenvalue problem to be obtained from Equations (57) and (58) will be as follows:

(59) $$\left[\begin{array}{cc} \phi_{11} & \phi_{12}\\ \phi_{21} & \phi_{22} \end{array}\right]\left[\begin{array}{c} \Psi_{0}\\ \Psi_{L} \end{array}\right]=0$$

The elements of the coefficient matrix given in Equation (59) are defined as:

(60) $$\phi_{11}=-\bar{\Omega_{0}}-1+\overset{\infty }{\underset{j=1}{\sum }}\frac{2\left(\lambda^{2}-K\right)}{-\lambda^{2}+K+\pi^{2}j^{2}}$$

(61) $$\phi_{12}=1-\overset{\infty }{\underset{j=1}{\sum }}\frac{2{\left(-1\right)}^{j}\left(+\lambda^{2}-K\right)}{-\lambda^{2}+K+\pi^{2}j^{2}}$$

(62) $$\phi_{21}=1-\overset{\infty }{\underset{j=1}{\sum }}\frac{2{\left(-1\right)}^{j}\left(+\lambda^{2}-K\right)}{-\lambda^{2}+K+\pi^{2}j^{2}}$$

(63) $$\phi_{22}=-\bar{\Omega_{L}}-1+\overset{\infty }{\underset{j=1}{\sum }}\frac{2\left(\lambda^{2}-K\right)}{-\lambda^{2}+K+\pi^{2}j^{2}}$$

The free vibration frequencies of embedded short-fiber-reinforced nano-rods are found by the eigenvalues of the coefficient matrix in Equation (59).

(64) $$\left|\phi_{\varphi \tau }\right|=0\;\left(\varphi ,\tau =1,2\right)$$

The above solution includes the impacts of the elastic medium parameter and axial spring parameters.

### 5.4. Without Elastic Medium and Size-Effect

To obtain the free axial frequencies of short-fiber-reinforced micro-/nano-rods without a size effect, the non-dimensional nonlocal parameter and elastic foundation parameter in Equations (39) and (40) are adjusted to zero. If we set the non-dimensional nonlocal parameter *β* and elastic foundation parameter *K* in Equations (39) and (40) to zero, we derive the following expressions:

(65) $$\left(-\bar{\Omega_{0}}-1+\overset{\infty }{\underset{j=1}{\sum }}\frac{2\lambda^{2}}{-\lambda^{2}+\pi^{2}j^{2}}\right)\Psi_{0}+\left(1-\overset{\infty }{\underset{j=1}{\sum }}\frac{2{\left(-1\right)}^{j}\lambda^{2}}{-\lambda^{2}+\pi^{2}j^{2}}\right)\Psi_{L}=0$$

(66) $$\left(1-\overset{\infty }{\underset{j=1}{\sum }}\frac{2{\left(-1\right)}^{j}\lambda^{2}}{-\lambda^{2}+\pi^{2}j^{2}}\right)\Psi_{0}+\left(-\bar{\Omega_{L}}-1+\overset{\infty }{\underset{j=1}{\sum }}\frac{2\lambda^{2}}{-\lambda^{2}+\pi^{2}j^{2}}\right)\Psi_{L}=0$$

With the help of Equations (64) and (65), the following eigenvalue problem is derived:

(67) $$\left[\begin{array}{cc} \Lambda_{11} & \Lambda_{12}\\ \Lambda_{21} & \Lambda_{22} \end{array}\right]\left[\begin{array}{c} \Psi_{0}\\ \Psi_{L} \end{array}\right]=0$$

The elements of the coefficient matrix given in Equation (67) are defined as:

(68) $$\Lambda_{11}=-\bar{\Omega_{0}}-1+\overset{\infty }{\underset{j=1}{\sum }}\frac{2\lambda^{2}}{-\lambda^{2}+\pi^{2}j^{2}}$$

(69) $$\Lambda_{12}=1-\overset{\infty }{\underset{j=1}{\sum }}\frac{2{\left(-1\right)}^{j}\lambda^{2}}{-\lambda^{2}+\pi^{2}j^{2}}$$

(70) $$\Lambda_{21}=1-\overset{\infty }{\underset{j=1}{\sum }}\frac{2{\left(-1\right)}^{j}\lambda^{2}}{-\lambda^{2}+\pi^{2}j^{2}}$$

(71) $$\Lambda_{22}=-\bar{\Omega_{L}}-1+\overset{\infty }{\underset{j=1}{\sum }}\frac{2\lambda^{2}}{-\lambda^{2}+\pi^{2}j^{2}}$$

The free vibration frequencies of short-fiber-reinforced classical rods are found by the eigenvalues of the coefficient matrix in Equation (67).

(72) $$\left|\Lambda_{\varphi \tau }\right|=0\;\left(\varphi ,\tau =1,2\right)$$

The above solution includes the effects of the axial spring parameters to examine the consequences of deformable boundary conditions on the axial vibration frequencies.

It should be noted here that there is wide-ranging, prominent knowledge on the effects of axial spring parameters. The main contribution of this study is that it presents the axial vibration behavior of embedded short-fiber-reinforced nano-rods with arbitrary boundary conditions. The boundary condition is one of the significant parameters affecting the vibration behavior of any element or structure. When looking at the boundary conditions studied in the literature, it is seen that most of them examine solutions performed under rigid boundaries (clamped at both ends or clamped–free for a nano-/micro-rod). In addition, the axial vibration behavior of short-fiber-reinforced nano-sized rods was examined by Gül and Aydoğdu [1] for the first time. Gül and Aydoğdu [1] considered clamped–clamped and clamped–free boundary conditions in their study. In the present paper, we examine the longitudinal vibration of short-fiber-reinforced nano-sized rods under arbitrary support conditions for the first time and include the impact of the elastic medium in the solutions.

## 6. Discussion

This section of the paper is dedicated to proving the correctness of the presented solution approach and presenting several numerical examples for the randomly oriented short-fiber-reinforced composite nano-rod. For this purpose, two comparison studies are first given for two different boundary conditions, with the results presented in the paper by Gül and Aydoğdu [1]. For these comparison studies, the material and geometrical properties of the composite nano-rod are considered as follows [1]: $\rho_{f}/\rho_{m}=4$, $E_{f}/E_{m}=10$, l/d=4, $V_{f}=0.5$ and L=20 nm. Table 1 compares the non-dimensional axial frequencies in the first three modes of the randomly oriented composite nano-rod with two ends clamped, while Table 2 compares the dimensionless axial vibration frequencies of the randomly oriented composite nano-rod with one end clamped and the other end free. Gül and Aydoğdu have not examined the effect of elastic medium in their study. Therefore, the elastic medium parameter *K* is set equal to zero in the comparison studies.

In the following part of the study, various numerical studies are performed for randomly oriented short-fiber-reinforced composite nano-sized rods with axial springs of infinite stiffness at both ends ($\bar{\Omega_{0}}=\bar{\Omega_{L}}=\infty$). Also, all calculations are done by j=50. These numerical studies are visualized with the help of a number of figures and the effects of various parameters are studied in detail. The frequencies examined in the study are in dimensionless form and the non-dimensional axial frequency of the composite nano-rod ($\bar{\lambda }$) is obtained in numerical studies as follows:

(73) $$\bar{\lambda }=\omega L\sqrt{\frac{\rho_{m}A}{E_{m}A}}$$

First, the impacts of the nonlocal parameter e_0_a on the dimensionless frequency values of the short-fiber-reinforced nano-rod are investigated. For this purpose, the dimensionless axial frequency values for nonlocal parameter values ranging from 0 nm to 0.5 nm are plotted for the first seven modes in Figure 3. The following properties are used for this figure: $\rho_{f}/\rho_{m}=4$, $E_{f}/E_{m}=10$, l/d=4, $V_{f}=0.5$ and L=20 nm. Also, the elastic medium effect is omitted in this example. When we look at the changes in the dimensionless axial frequencies of the short-fiber-reinforced composite nano-rod with the help of the figure, we can say that a general decrease has occurred. In the first mode, when the nonlocal parameter is 0.0 nm, 0.1 nm, 0.2 nm and 0.3 nm, or when the nonlocal parameter is 0.0 nm and 0.1 nm in the second mode, there is no change in the dimensionless axial frequencies. This can be easily explained by the impact of the nonlocal parameter on the vibrational modes. It should be noted that the amount of the decrease in the non-dimensional frequencies increases with the increase in the vibration mode number. In the first and second modes the changes are especially negligible, while in the higher modes the differences become more pronounced. Thus, it can be concluded that the impact of the nonlocal parameter on the axial frequencies of the short-fiber-reinforced composite nano-rod in higher modes is more significant.

**Figure 4:**
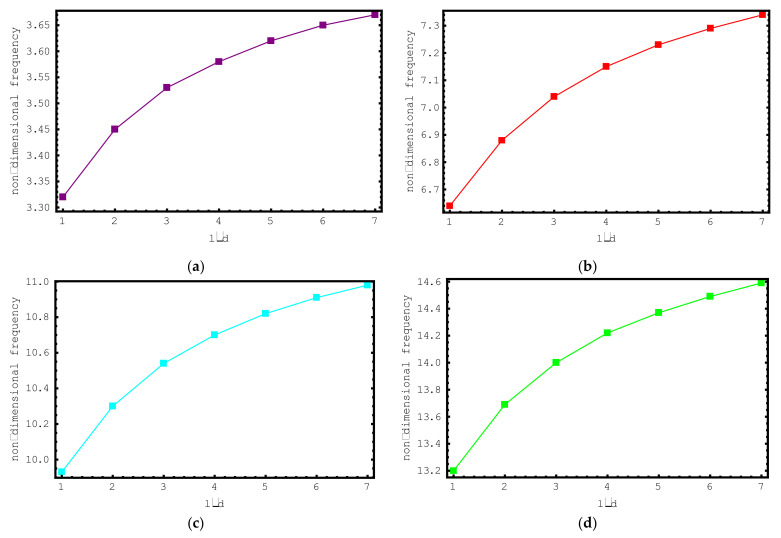

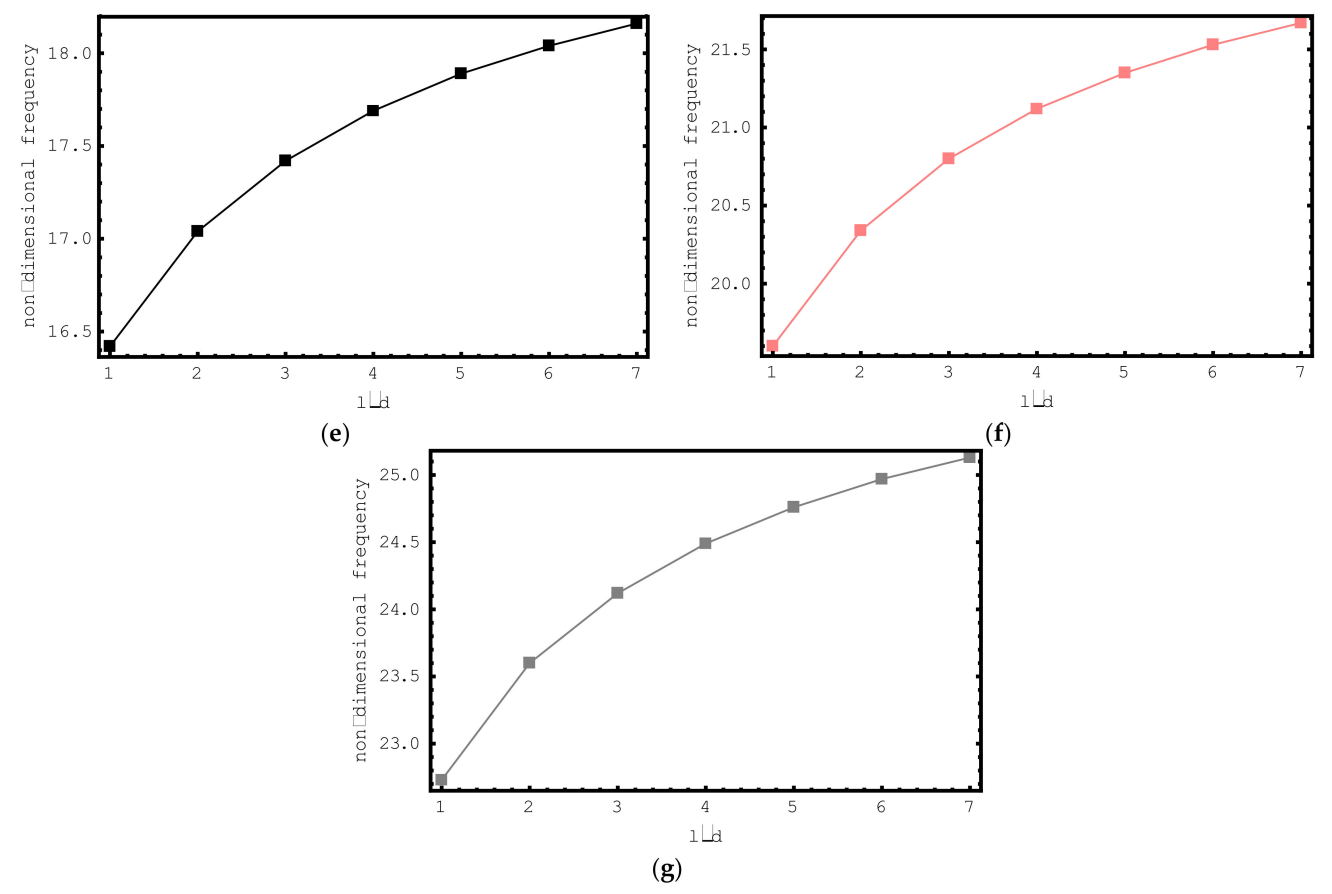
The variations of non-dimensional frequencies of short-fiber-reinforced composite nano-rods versus *l*/*d*: (**a**) 1st mode (**b**) 2nd mode (**c**) 3rd mode (**d**) 4th mode (**e**) 5th mode (**f**) 6th mode (**g**) 7th mode.

Secondly, the impacts of l/d ratios on the dimensionless frequency values of the short-fiber-reinforced nano-rod are examined. For this purpose, non-dimensional axial frequency values for l/d values ranging from one to seven are illustrated for the first seven modes via Figure 4. The following properties are utilized for this investigation: $\rho_{f}/\rho_{m}=4$, $E_{f}/E_{m}=10$, $e_{0}a=0.2\;\mathrm{nm}$, $V_{f}=0.5$, K=0 and L=20 nm. It can be clearly seen from Figure 4 that with increasing l/d values, the dimensionless frequency values of the short-fiber-reinforced nano-rod also increase. This increment is valid for all modes examined. It should be noted that at low l/d values, the change in the dimensionless frequencies of the short-fiber-reinforced composite nano-rod is more significant. As the l/d values increase, the change in the dimensionless frequencies decreases.

**Figure 5:**
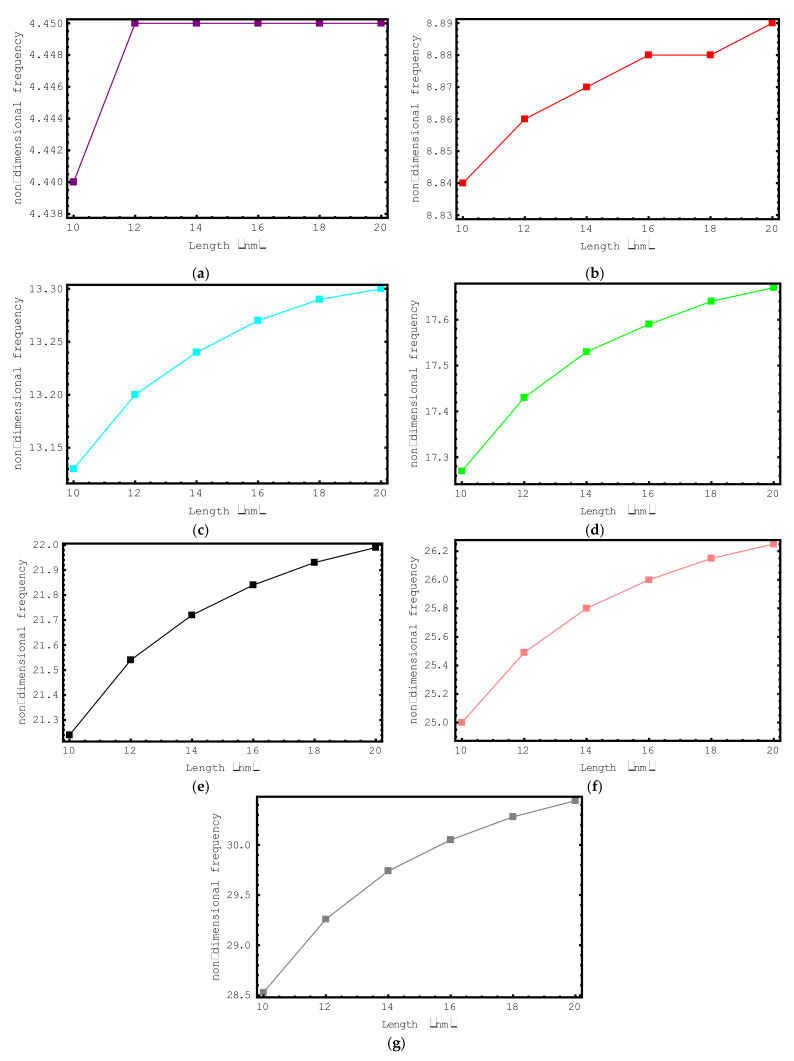
The variations of non-dimensional frequencies of short-fiber-reinforced composite nano-rods versus length: (**a**) 1st mode (**b**) 2nd mode (**c**) 3rd mode (**d**) 4th mode (**e**) 5th mode (**f**) 6th mode (**g**) 7th mode.

Via Figure 5, the influence of composite nano-rod length on the dimensionless frequency values is discussed. In Figure 5, the variation in the non-dimensional axial frequency values of short-fiber-reinforced composite nano-rods versus length is plotted for the first seven modes. The length of the composite nano-rod ranges from 10 nm to 20 nm and the following properties are considered: $\rho_{f}/\rho_{m}=2$, $E_{f}/E_{m}=10$, $e_{0}a=0.2\;\mathrm{nm}$, $V_{f}=0.5$, K=0 and l/d=2. When we look at the changes in the dimensionless axial frequencies of the short-fiber-reinforced composite nano-rod via the figure, we can say that a general increase has occurred. In the first mode, when the length is 12 nm, 14 nm, 16 nm, 18 nm and 20 nm, there is no change in the dimensionless axial frequencies of the composite nano-rod. This can be explained by the impact of the length on the vibrational modes. It should be highlighted here that the amount of the increment in the non-dimensional frequencies increases with the increase in the vibration mode number. It may be said that the impact of nano-rod length on the non-dimensional frequencies in higher modes is more prominent.

**Figure 6:**
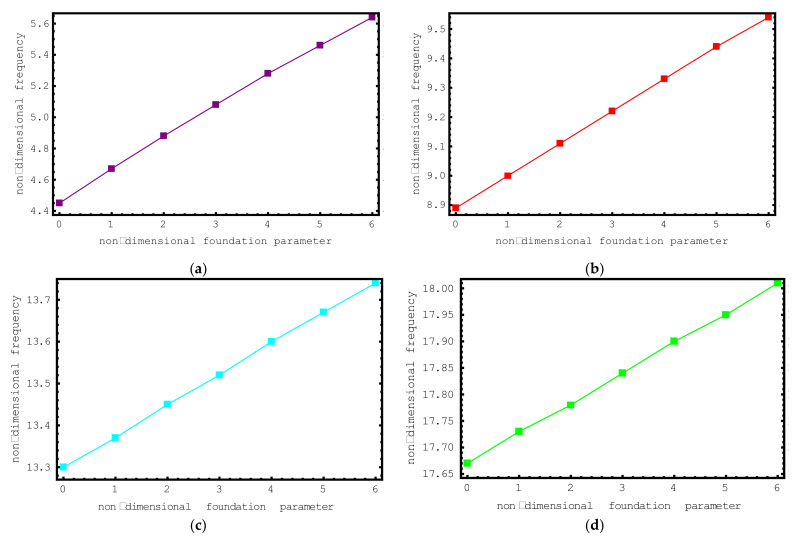

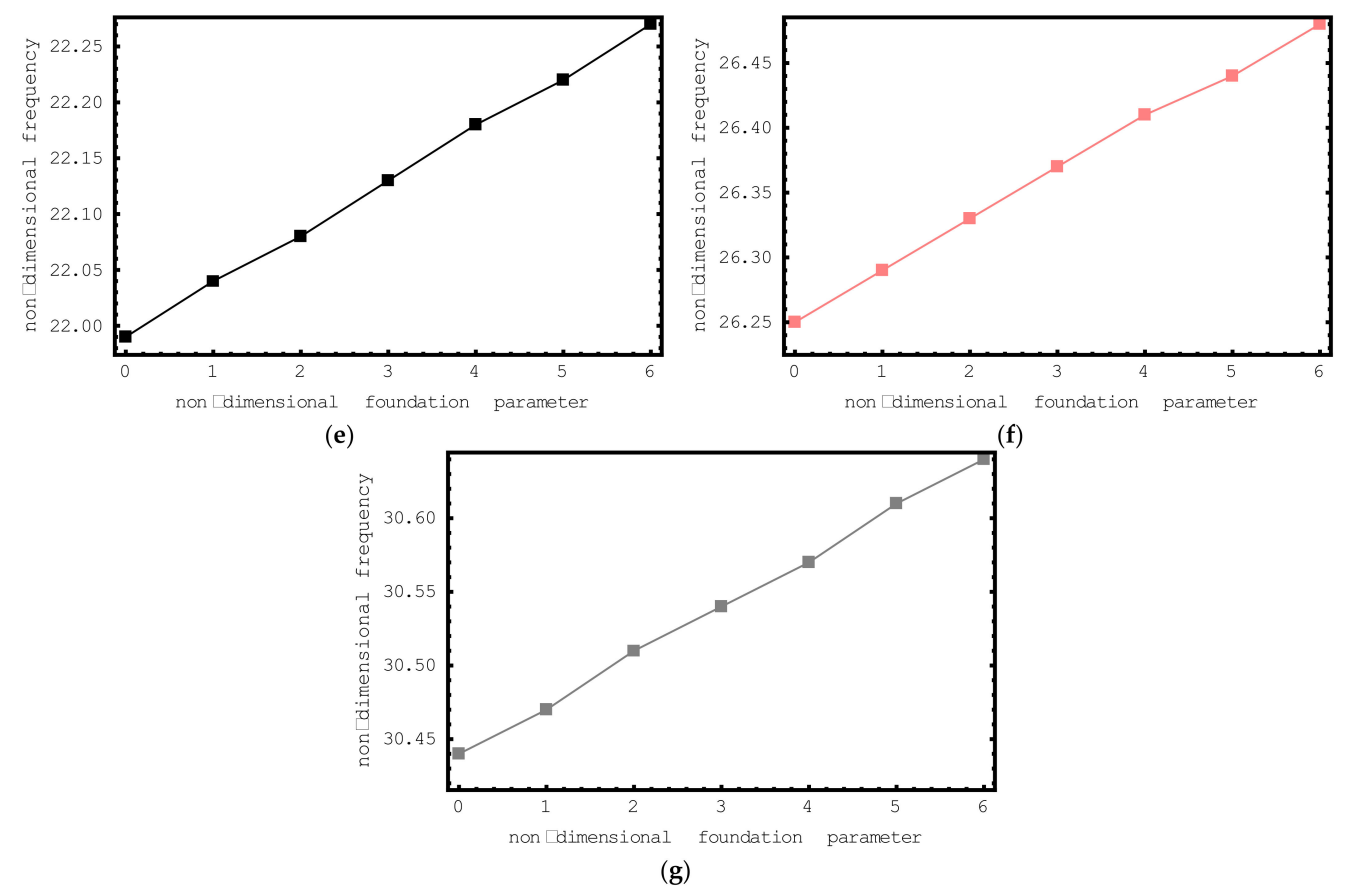
The variations of non-dimensional frequencies of short-fiber-reinforced composite nano-rods versus the foundation parameter: (**a**) 1st mode (**b**) 2nd mode (**c**) 3rd mode (**d**) 4th mode (**e**) 5th mode (**f**) 6th mode (**g**) 7th mode.

In Figure 6, the influence of elastic foundation is investigated. For this aim, the dimensionless frequency values of the short-fiber-reinforced composite nano-rod are plotted against the dimensionless foundation parameter *K* for the first seven modes. The dimensionless foundation parameter impacting the composite nano-rod changes from zero to six and the following properties are considered in this investigation: $\rho_{f}/\rho_{m}=2$, $E_{f}/E_{m}=10$, $e_{0}a=0.2\;\mathrm{nm}$, $V_{f}=0.5$, L=20 nm and l/d=2. From this figure, the increase in the dimensionless frequencies of the short-fiber-reinforced nano-sized rod with increasing foundation parameter values can be clearly discerned. It should be noted here that if the dimensionless foundation parameter *K* is set to zero, the composite nano-sized rod becomes independent of the foundation effect. It should also be noted that the lowest frequency values are calculated at *K* = 0. From this it is clear that the presence of an elastic medium has a hardening effect on the short-fiber-reinforced composite nano-rod. Another important issue to be emphasized here is the influence of elastic foundation on the vibration modes. When Figure 6 is examined, it can be seen that the increases in the dimensionless frequency values are much higher in the first mode. If the modes are analyzed separately, it is clear that the lowest amount of change occurs in the seventh mode. Based on these results, it is possible to say that the elastic foundation effect is much more effective in lower modes.

**Figure 7:**
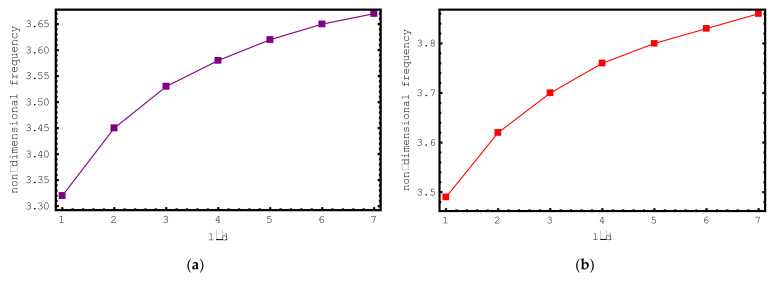

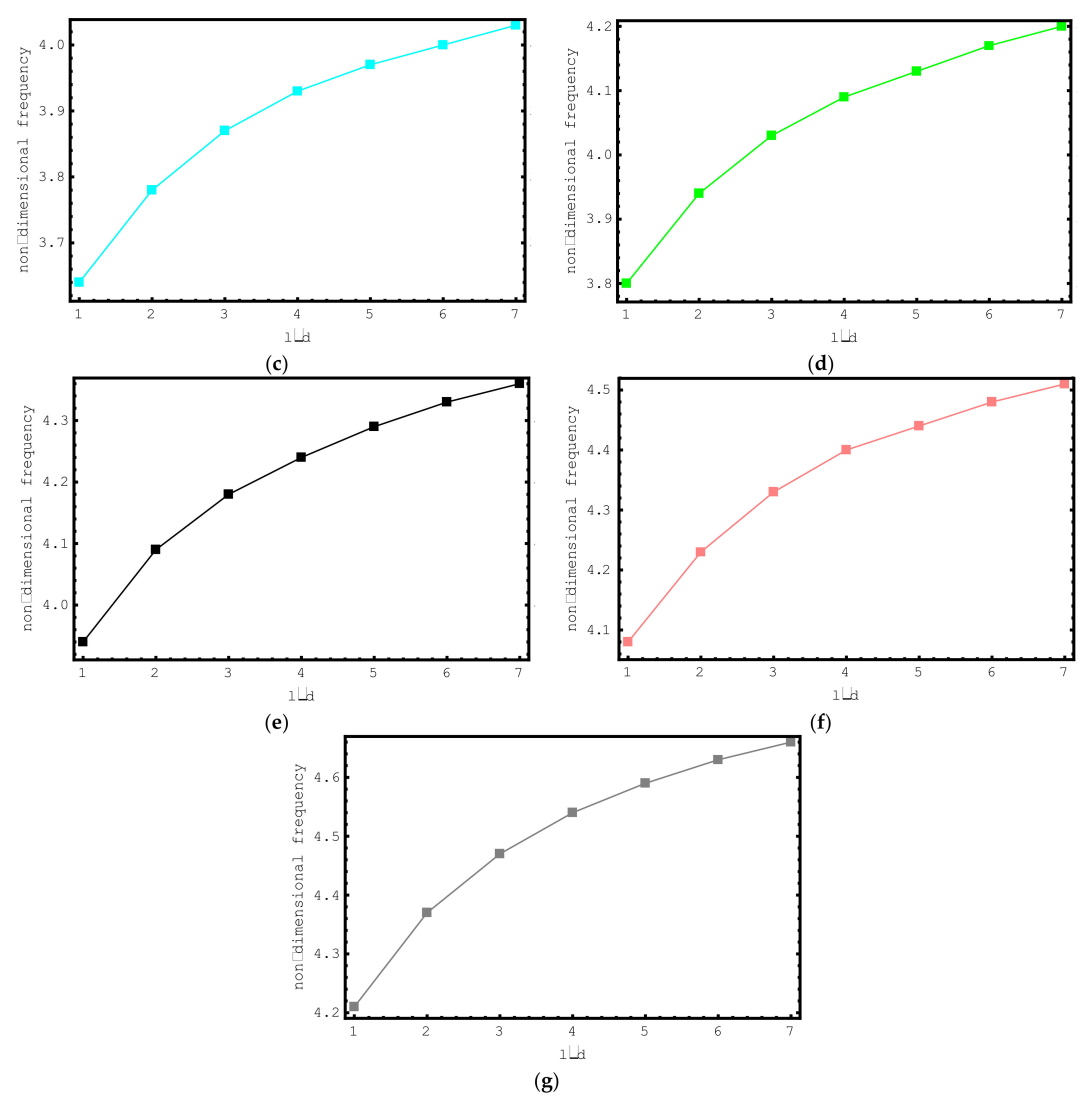
The variations of non-dimensional frequencies of short-fiber-reinforced composite nano-rods versus *l*/*d*: (**a**) *K* = 0 (**b**) *K* = 1 (**c**) *K* = 2 (**d**) *K* = 3 (**e**) *K* = 4 (**f**) *K* = 5 (**g**) *K* = 6.

Figure 7 aims to investigate the impacts of the foundation parameter and *l*/*d* ratio on the dimensionless frequencies of the short-fiber-reinforced composite nano-rod. For this purpose, Figure 7 demonstrates the alteration of the first mode dimensionless frequency values of the composite nano-rod versus *l*/*d* for various foundation parameters *K*. The dimensionless foundation parameter and *l*/*d* are changed from zero to six and from one to seven, respectively. In addition, the following properties are used in this investigation: $\rho_{f}/\rho_{m}=4$, $E_{f}/E_{m}=10$, $e_{0}a=0.2\;\mathrm{nm}$, $V_{f}=0.5$, L=20 nm and l/d=2. From here, the increase in frequencies caused by the foundation parameter and *l*/*d* can be observed again.

**Figure 8:**
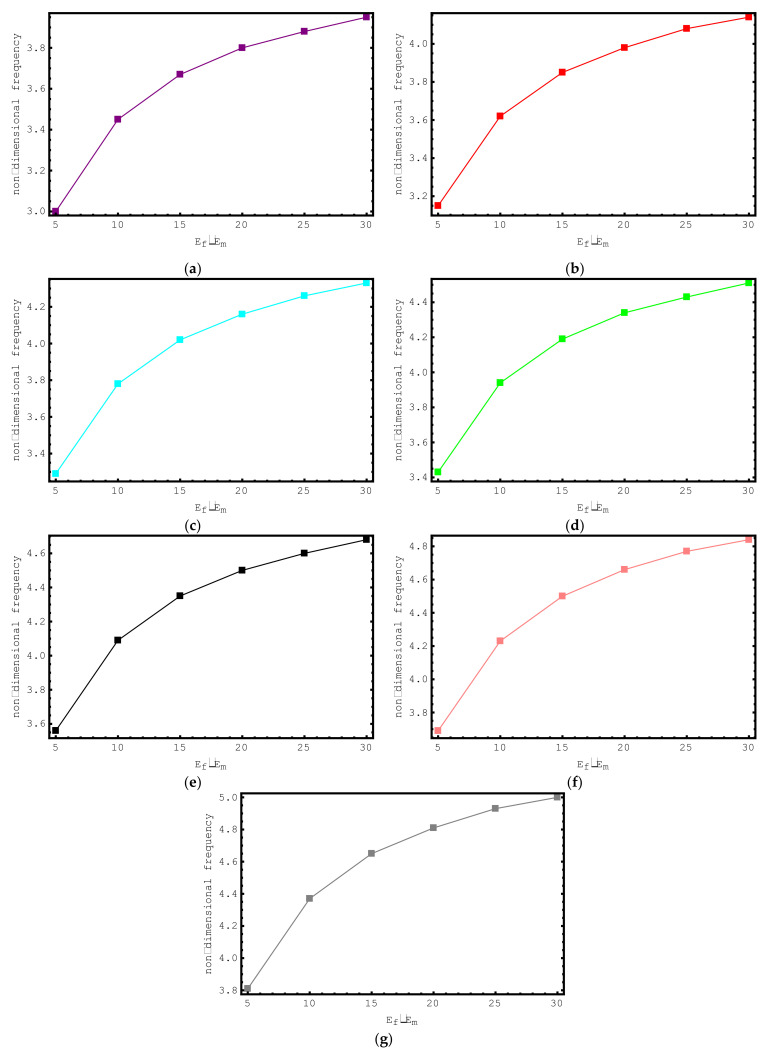
The variations of non-dimensional frequencies of short-fiber-reinforced composite nano-rods versus E_f_/E_m_: (**a**) *K* = 0 (**b**) *K* = 1 (**c**) *K* = 2 (**d**) *K* = 3 (**e**) *K* = 4 (**f**) *K* = 5 (**g**) *K* = 6.

Figure 8 demonstrates the impacts of the foundation parameter and the *E_f_*/*E_m_* ratio on the first dimensionless frequencies of the embedded short-fiber-reinforced composite nano-rod. In this figure, the change in the first mode dimensionless frequencies of the composite nano-rod versus *E_f_*/*E_m_* for various foundation parameters *K* is plotted. The dimensionless foundation parameter and *E_f_*/*E_m_* are changed from zero to six and from 5 to 30, respectively. Furthermore, the following properties are assumed for this figure: $\rho_{f}/\rho_{m}=4$, l/d=2, $e_{0}a=0.2\;\mathrm{nm}$, $V_{f}=0.5$, L=20 nm. It can be understood from this figure that an increment in the *E_f_*/*E_m_* value is accompanied by an increase in the first-mode axial frequencies.

**Figure 9:**
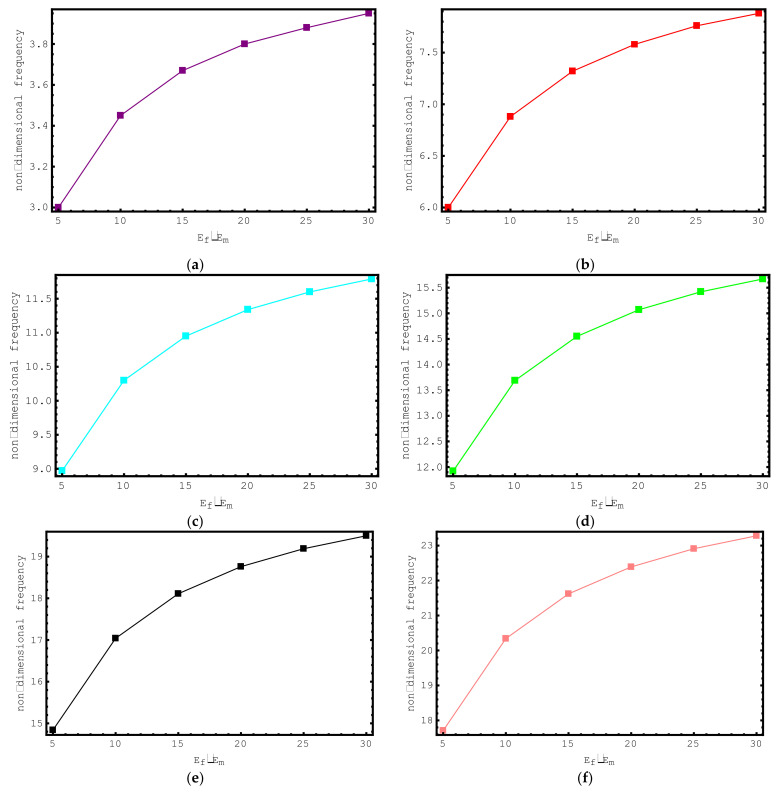

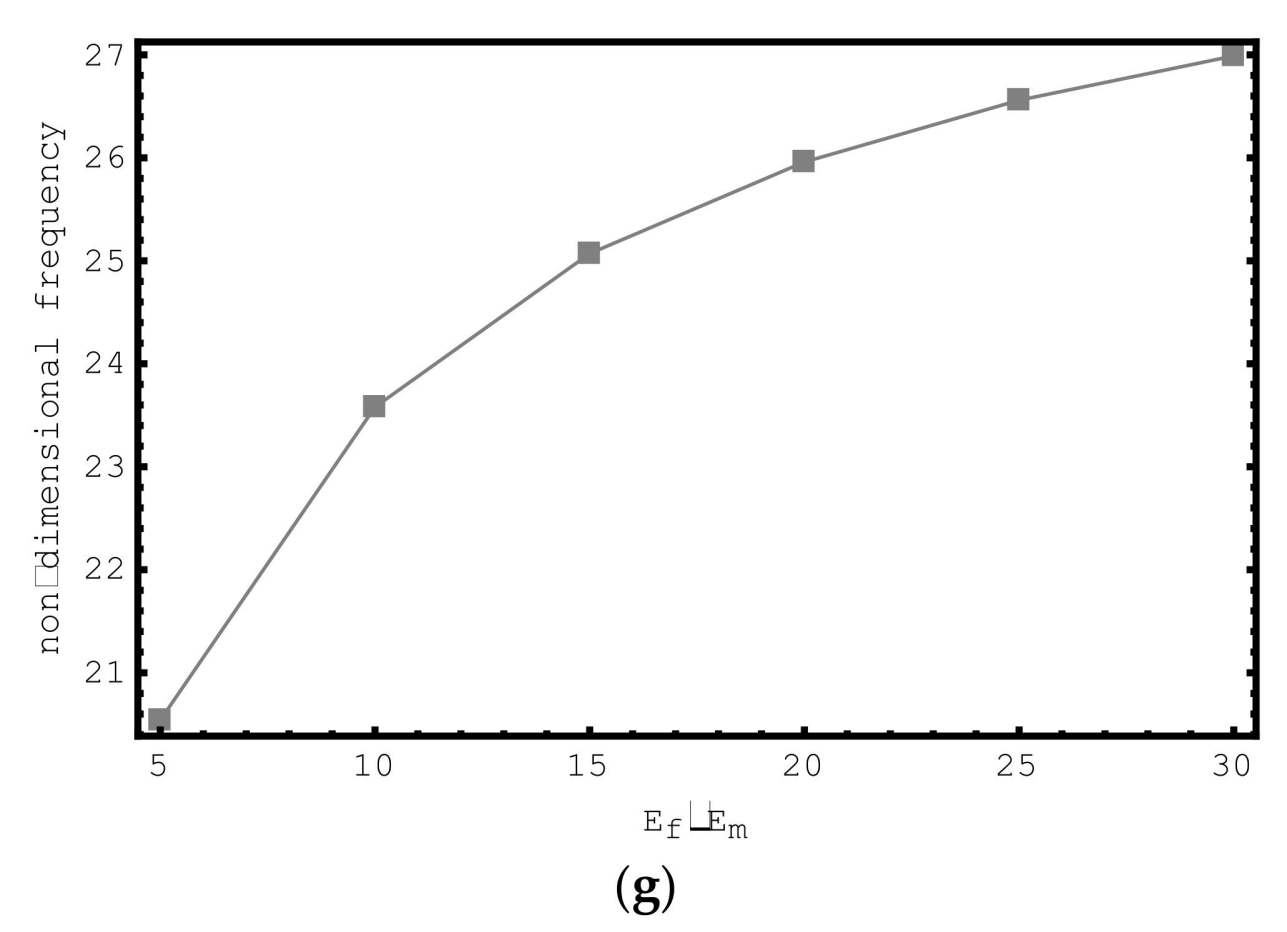
The variations of non-dimensional frequencies of short-fiber-reinforced composite nano-rods versus E_f_/E_m_: (**a**) 1st mode (**b**) 2nd mode (**c**) 3rd mode (**d**) 4th mode (**e**) 5th mode (**f**) 6th mode (**g**) 7th mode.

The influence of *E_f_*/*E_m_* ratios on the dimensionless frequency values of the short-fiber-reinforced nano-rod are examined in Figure 9. For this purpose, the non-dimensional axial frequency values for *E_f_*/*E_m_* values ranging from 5 to 30 are demonstrated for the first seven modes. The following properties are considered for this study: $\rho_{f}/\rho_{m}=4$, $e_{0}a=0.2\;nm$, $V_{f}=0.5$, K=0, l/d=2, and L=20 nm. It is clearly seen here that with increasing *E_f_*/*E_m_* values, the dimensionless frequencies of the composite nano-sized rod increase. It should be emphasized here that at low *E_f_*/*E_m_* values, the change in the dimensionless axial frequencies of the short-fiber-reinforced nano-rod is more prominent. As the *E_f_*/*E_m_* values increase, the change in the dimensionless axial frequencies of the composite nano-rod decreases.

**Figure 10:**
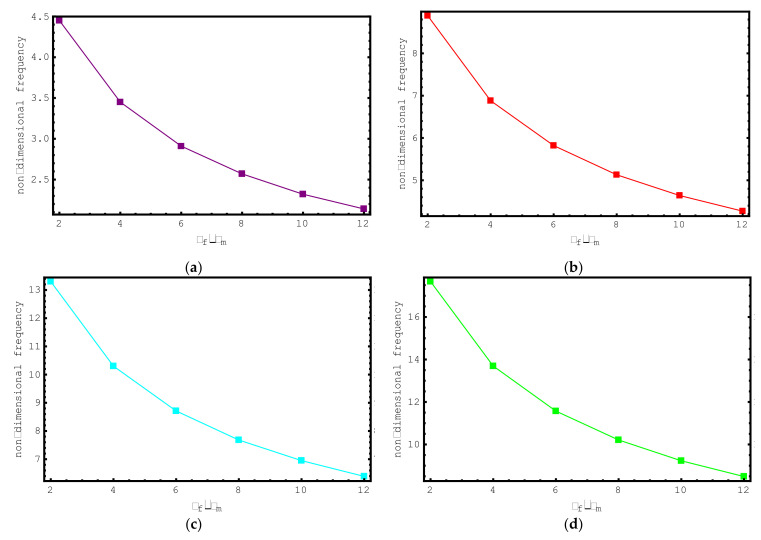

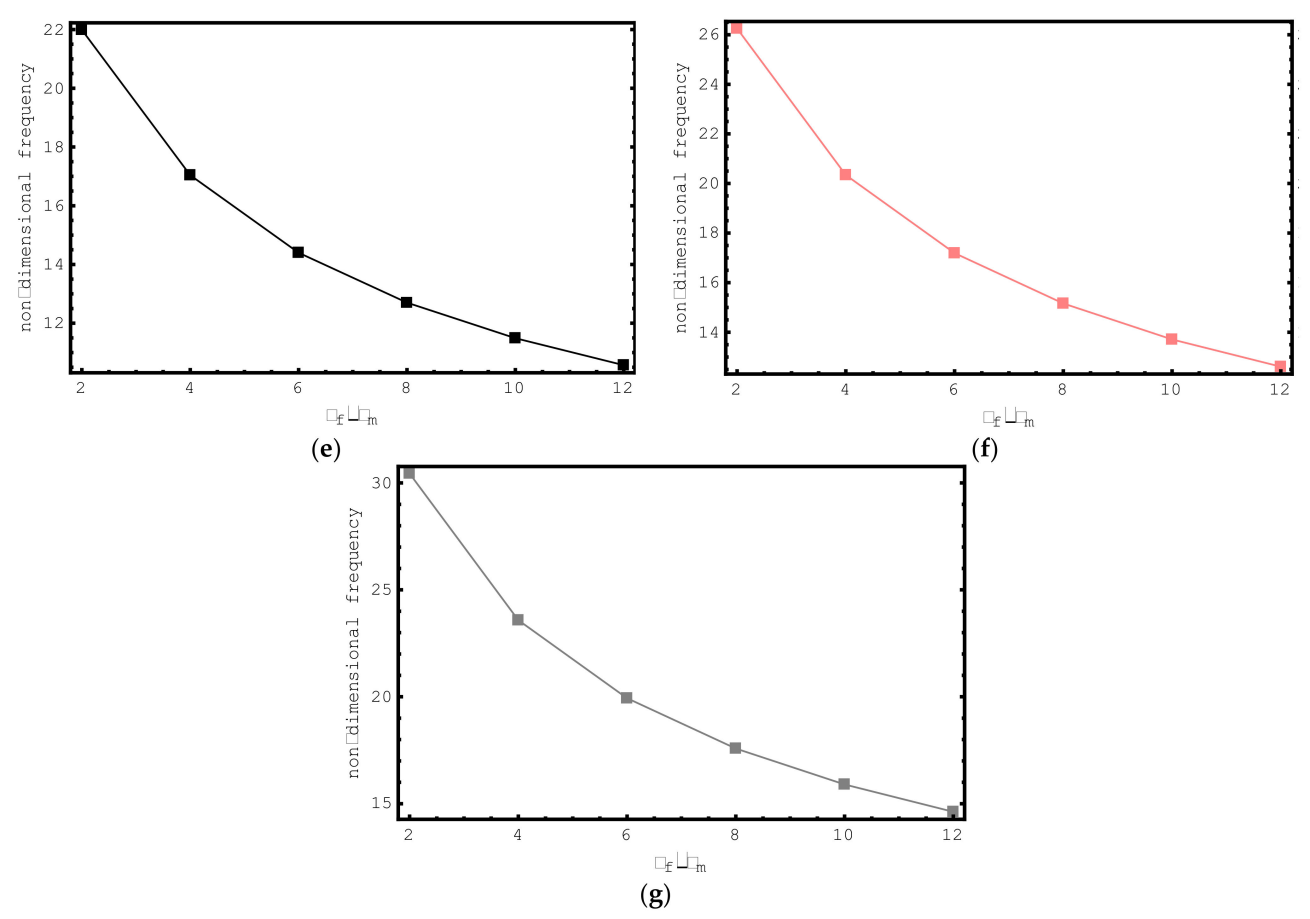
The variations of non-dimensional frequencies of short-fiber-reinforced composite nano-rods versus ***ρ***_f_/***ρ***_m_: (**a**) 1st mode (**b**) 2nd mode (**c**) 3rd mode (**d**) 4th mode (**e**) 5th mode (**f**) 6th mode (**g**) 7th mode.

Lastly, the impacts of *ρ_f_*/*ρ_m_* ratios on the dimensionless frequencies of the composite nano-sized rod are examined in Figure 10. For this purpose, non-dimensional axial frequency values for *ρ_f_*/*ρ_m_* values ranging from 2 to 12 are shown for the first seven modes. The following properties are considered for this study: $E_{f}/E_{m}=10$, $e_{0}a=0.2\;nm$, $V_{f}=0.5$, K=0, l/d=2, and L=20 nm. It is observed here that with increasing *ρ_f_*/*ρ_m_* values, the non-dimensional frequency values of the short-fiber-reinforced composite nano-rod decrease. This decrement in the frequencies is valid for all modes examined. It should be highlighted here that at low *ρ_f_*/*ρ_m_* values, the variation in the dimensionless axial frequencies of the short-fiber-reinforced nano-rod is more conspicuous.

## 7. Conclusions

In this paper, the dynamics of embedded short-fiber-reinforced micro-/nano-rods have been investigated using Eringen’s nonlocal elasticity theory. Based on this higher-order theory and the material properties of the nano-rods, a coefficient matrix including the elastic foundation, short fiber and the nonlocal parameter are obtained. The systems of linear equations including infinite power series are constructed by introducing the nonlocal force boundary conditions and with the help of the Stokes’ transformation together with Fourier sine series. A precise and constant eigenvalue algorithm is applied to obtain the axial vibration frequencies of composite nano-rods with deformable and rigid boundary conditions. The present model is validated by comparing the analytical results with the results available in the scientific literature. Eringen’s nonlocal small-scale parameter has a softening effect on the free axial vibration frequencies for all the boundary conditions (rigid or restrained), without observing the paradoxical response of nonlocal elasticity theory. The hardening effect of the short-fiber parameter is more pronounced for all the boundary conditions. Similar studies for other behaviors of short-fiber-reinforced nano-rods, such as buckling, bending, wave propagation and forced vibration propagation, can be conducted in future works.

## Figures and Tables

**Table 1:** Comparison of the first three non-dimensional axial frequencies of randomly oriented short-fiber-reinforced composite nano-rods for the clamped–clamped boundary condition.

Mode Number	Analytical Solution [1]	Present $\bar{(\Omega_{0}}=\bar{\Omega_{L}}=\infty )$
*e*_0_*a* = 0 nm		
1	3.5819	3.5819
2	7.1639	7.1639
3	10.7459	10.7459
*e*_0_*a* = 0.5 nm		
1	3.5709	3.5709
2	7.0771	7.0771
3	10.4594	10.4594
*e*_0_*a* = 1 nm		
1	3.5385	3.5385
2	6.8345	6.8345
3	9.7206	9.7206

**Table 2:** Comparison of the first three non-dimensional axial frequencies of randomly oriented short-fiber-reinforced composite nano-rods for the clamped–free boundary condition.

Mode Number	Analytical Solution [1]	Present $\bar{(\Omega_{0}}=\infty ,\;\bar{\;\Omega_{L}}=0)$
*e*_0_*a* = 0 nm		
1	1.7909	1.7909
2	5.3729	5.3729
3	8.9549	8.9549
*e*_0_*a* = 0.5 nm		
1	1.7896	1.7896
2	5.3360	5.3360
3	8.7871	8.7871
*e*_0_*a* = 1 nm		
1	1.7854	1.7854
2	5.2297	5.2297
3	8.3352	8.3352
